# Validation and verification of predictive salivary biomarkers for oral health

**DOI:** 10.1038/s41598-021-85120-w

**Published:** 2021-03-19

**Authors:** Nagihan Bostanci, Konstantinos Mitsakakis, Beral Afacan, Kai Bao, Benita Johannsen, Desirée Baumgartner, Lara Müller, Hana Kotolová, Gülnur Emingil, Michal Karpíšek

**Affiliations:** 1 Section of Periodontology and Dental Prevention, Division of Oral Diseases, Department of Dental Medicine, Karolinska Institutet, Alfred Nobels Allé 8, 14104 Huddinge, Stockholm, Sweden; 2 Hahn-Schickard, Georges-Koehler-Allee 103, 79110 Freiburg, Germany; 3 Laboratory for MEMS Applications, IMTEK – Department of Microsystems Engineering, University of Freiburg, Georges-Koehler-Allee 103, 79110 Freiburg, Germany; 4 Department of Periodontology, School of Dentistry, Aydin University, Hasan Efendi mah. Hastane cad. No:1 Efeler/Aydın, 09100 Istanbul, Turkey; 5 Faculty of Pharmacy, Masaryk University, Palackeho trida 1946/1, 61242 Brno, Czech Republic; 6 Department of Periodontology, School of Dentistry, Ege University, 35100 Bornova, İzmir Turkey; 7 BioVendor-Laboratorní Medicína a.s., Research and Diagnostic Products Division, Karasek 1767/1, Reckovice, 62100 Brno, Czech Republic

**Keywords:** Gingivitis, Periodontitis, Diagnostic markers

## Abstract

Oral health is important not only due to the diseases emerging in the oral cavity but also due to the direct relation to systemic health. Thus, early and accurate characterization of the oral health status is of utmost importance. There are several salivary biomarkers as candidates for gingivitis and periodontitis, which are major oral health threats, affecting the gums. These need to be verified and validated for their potential use as differentiators of health, gingivitis and periodontitis status, before they are translated to chair-side for diagnostics and personalized monitoring. We aimed to measure 10 candidates using high sensitivity ELISAs in a well-controlled cohort of 127 individuals from three groups: periodontitis (60), gingivitis (31) and healthy (36). The statistical approaches included univariate statistical tests, receiver operating characteristic curves (ROC) with the corresponding Area Under the Curve (AUC) and Classification and Regression Tree (CART) analysis. The main outcomes were that the combination of multiple biomarker assays, rather than the use of single ones, can offer a predictive accuracy of > 90% for gingivitis versus health groups; and 100% for periodontitis versus health and periodontitis versus gingivitis groups. Furthermore, ratios of biomarkers MMP-8, MMP-9 and TIMP-1 were also proven to be powerful differentiating values compared to the single biomarkers.

## Introduction

Periodontal disease is an inflammatory disease that destroys the tooth-supporting tissues^1^. The diagnosis of the disease largely relies on clinical and radiographic examinations that assess previously occurring tissue damage. Hence, the current diagnostic protocols do not hold adequate sensitivity to help identify the active phase of disease or individuals at risk for active disease^2^. Thus, measurable biological indicators with higher sensitivity and accuracy are needed in order to place the diagnosis of the disease on molecular evidence^3,4^.

Saliva is a favorable oral fluid to determine the health state of the oral cavity, including the presence of periodontal disease^5–7^. Several methods have been used in the analysis of saliva, including immunoassays, colorimetric methods and most recently quantitative mass spectrometric methods. Although more than 5000 proteins were identified in saliva, not all have been further validated and only a few have been investigated in more detail^8–11^. Despite some protein biomarkers have been marketed for periodontal disease monitoring^12,13^, most of them disappeared from the market very fast due to their low specificity^14^. The presence of an individual protein is not adequate to capture the episodic progression of the disease or differentiate stages of periodontal disease^3,15–17^. As diagnostic tests based on combinations of markers promise improved performance over those on a single marker, the present cross-sectional cohort study aims to compare the salivary concentrations of 10 candidate proteins (selected based on their relevance to periodontal disease) in individuals with a clinical diagnosis of periodontal health, gingivitis or periodontitis. Since antibody-based ELISA assays are still the gold standard for detecting multiple proteins in body fluids, including saliva^10,18,19^, the present study was conducted on this basis. The 10 candidate proteins are: matrix metalloproteinases 8, 9, 2, 3 (MMP-8, MMP-9, MMP-2, MMP-3); tissue inhibitor of metalloproteinases (TIMP-1); osteoprotegerin (OPG); interleukins 1 beta and 8 (IL-1b, IL-8); hepatocyte growth factor (HGF); and lipopolysaccharide-binding protein (LBP). The value of each candidate for diagnostic prediction was assessed by (i) univariate statistical tests; (ii) estimation of the Receiver Operating Characteristic (ROC) curves and the Area Under the Curve (AUC); and (iii) Classification and Regression Tree Analysis (CART). Consequently, the intended use of such a multi-marker test is the identification of the most promising biomarkers and their combination for the most efficient molecular-based differentiation between healthy, periodontitis and gingivitis individuals. Thus, the clinical role of future implementation of these biomarkers in the clinical practice is related to (i) the early diagnosis of the aforementioned conditions, contributing to disease prevention; and (ii) the precise monitoring of the improving health status during relevant periodontal treatment.

## Results

### Clinical and demographic characteristics of the studied groups

A total of 127 saliva samples were assessed which were obtained from 36 individuals in the health group, 31 individuals in the gingivitis group and 60 subjects in the periodontitis group. All subjects were non-smokers. The demographic and clinical details of the participants included in the analysis are presented in Table 1.

**Table 1:** Demographics and full-mouth clinical parameters of the study groups.

Patients characteristics	Periodontitis (n = 60)	Gingivitis (n = 31)	Health (n = 36)
**Demographic variables**			
Age (years; mean ± SD)	39.6 ± 5.7*	33.1 ± 5.9	33.7 ± 6.7
**Sex (n)**			
Females	33	18	20
Males	27	13	16
**Periodontal parameters**			
PPD (mm)	4.0 ± 0.8*	2.3 ± 0.2	1.7 ± 0.2
CAL (mm)	5.1 ± 1.1*	0	0
BOP	86.4 ± 10.4*	68.7 ± 12.9	1.6 ± 0.9
PI	3.5 ± 0.6*	2.9 ± 0.5	1.8 ± 0.3

All data are given in terms of mean ± SD. PPD: Probing Pocket Depth. CAL Clinical Attachment Loss. BOP Bleeding On Probing. PI Plaque Index.

*p < 0.05: significantly higher than the gingitivis and health groups.

### Biomarker analytical results

Based on the ELISA measurements, the limit of detection (LOD) as well as intra- and inter-assay coefficient of variation (CV) were calculated for all markers (Table 2). Importantly, all CVs were below 10%, which allows a quantitative analysis of the assay results and ensures the reliability of data for the subsequent statistical analysis.

**Table 2:** Limit of Detection (LOD), intra/inter-assay coefficient of variation (CV) of the examined biomarker ELISA assays.

Measured biomarker	LOD	Intra-assay CV (%)	Inter-assay CV (%)
MMP-8	0.025 ng/ml	3.2	7.5
MMP-9	0.01 ng/ml	5.2	7.8
TIMP-1	0.05 ng/ml	2.4	6.2
LBP	0.03 ng/ml	6.2	9.8
MMP-2	0.01 ng/ml	4.5	6.6
MMP-3	0.1 ng/ml	4.6	8.8
OPG	0.03 pmol/l	3.5	5.8
IL-1b	0.4 pg/ml	3.5	6.0
IL-8	0.5 pg/ml	4.4	7.2
HGF	10 pg/ml	6.3	9.2

### Univariate statistical tests

**Figure 1:**
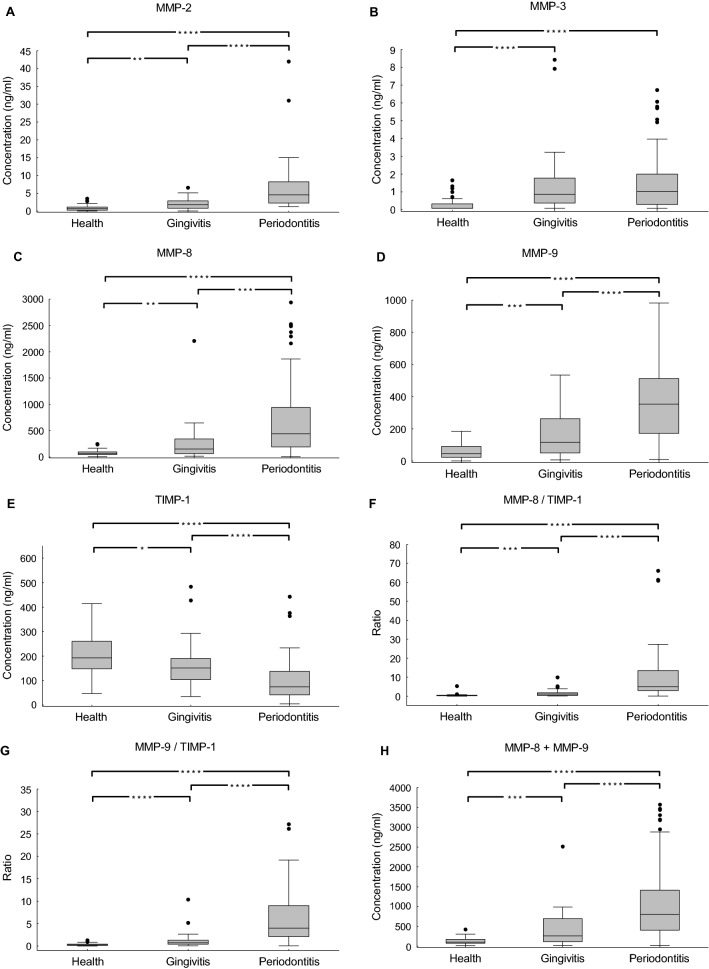

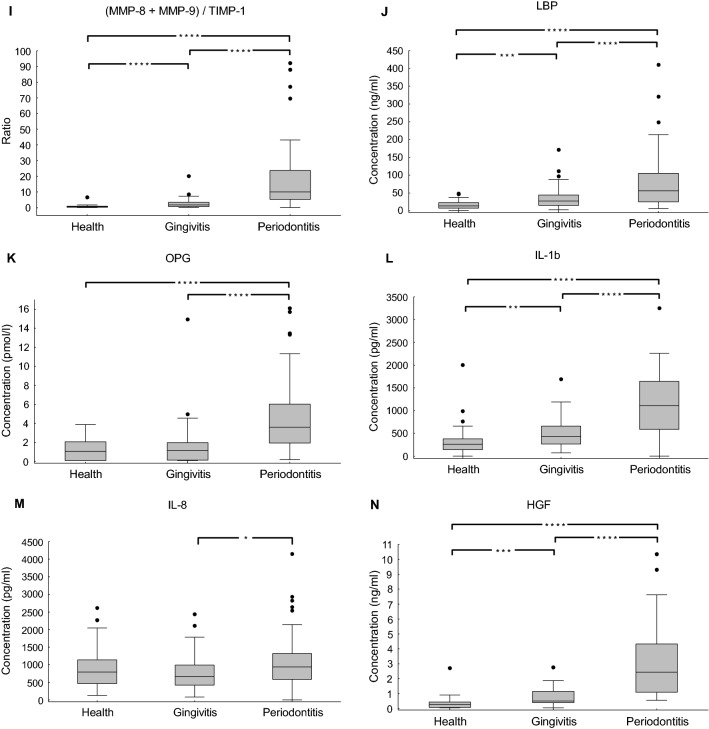
Box plots of the selected markers (and combinations thereof) showing levels of each in saliva from individuals in a comparison between health, gingivitis and periodontitis groups: (**A**) MMP-2; (**B**) MMP-3; (**C**) MMP-8; (**D**) MMP-9; (**E**) TIMP-1; (**F**) MMP-8/TIMP-1; (**G**) MMP-9/TIMP-1; (**H**) MMP-8 + MMP-9; (**I**) (MMP-8 + MMP-9)/TIMP-1; (**J**) LBP; (**K**) OPG; (**L**) IL-1b; (**M**) IL-8; (**N**) HGF. The boxes represent the values from the 25^th^ to the 75^th^ percentile. The middle lines represent the medians. The vertical lines extend from the minimal to the maximal values. (*) for *p* < 0.05; (**) for *p* < 0.01; (***) for *p* < 0.001; (****) for *p* < 0.0001.

We first assessed if there were any significant differences between healthy individuals and patients with gingivitis or periodontitis. The boxplot graphs of the studied salivary markers are displayed in Fig. 1. The mean and median concentrations of each marker for all three examined groups as well as the comparison between the groups are provided in Table 3.

**Table 3:** Mean and median concentrations of each marker for the three examined groups.

Biomarkers or combinations thereof	Unit	Health (H) (N = 36)			Gingivitis (G) (N = 31)			Periodontitis (P) (N = 60)			(H) vs (G)	(H) vs (P)	(G) vs (P)	Ratios between different groups		
		Mean	Median	SD	Mean	Median	SD	Mean	Median	SD	*p* values			Ratio G/H	Ratio P/H	Ratio P/G
MMP-8	ng/ml	76.5	65.8	53.7	272.4	150.5	403.2	740.7	439.8	783.0	0.0012	< 0.0001	0.0002	3.56	9.69	2.72
MMP-9	ng/ml	57.0	46.2	42.2	162.8	117.0	141.5	423.4	353.8	327.1	0.0001	< 0.0001	< 0.0001	2.86	7.43	2.60
TIMP-1	ng/ml	209.3	192.7	80.0	180.6	151.4	124.2	101.5	74.3	90.8	0.0089	< 0.0001	0.0001	0.86	0.49	0.56
Ratio: MMP-8 / TIMP-1	no units^#^	0.49	0.34	0.85	1.68	1.11	2.01	16.51	5.01	50.21	0.0001	< 0.0001	< 0.0001	3.42	33.69	9.85
Ratio: MMP-9 / TIMP-1	no units^#^	0.320	0.225	0.283	1.305	0.723	1.973	8.916	4.001	16.029	< 0.0001	< 0.0001	< 0.0001	4.08	27.90	6.83
Sum: MMP-8 + MMP-9	ng/ml	133.5	106.0	87.8	435.2	258.3	489.0	1164.0	802.2	1056.9	0.0003	< 0.0001	< 0.0001	3.26	8.72	2.67
Ratio: (MMP-8 + MMP-9) / TIMP-1	no units^#^	0.81	0.53	1.07	2.98	1.77	3.82	25.43	10.02	64.04	< 0.0001	< 0.0001	< 0.0001	3.68	31.39	8.53
LBP	ng/ml	16.258	13.823	11.410	39.387	27.347	36.791	79.590	56.091	79.329	0.0004	< 0.0001	0.0058	2.42	4.90	2.02
MMP-2	ng/ml	0.883	0.670	0.790	2.033	1.800	1.550	6.503	4.565	6.849	0.0006	< 0.0001	< 0.0001	2.30	7.37	3.20
MMP-3	ng/ml	0.294	0.080	0.398	1.461	0.860	1.961	1.558	1.025	1.646	< 0.0001	< 0.0001	0.5577	4.97	5.30	1.07
OPG	pmol/l	1.196	1.045	1.071	1.759	1.160	2.733	4.539	3.585	3.677	0.6020	< 0.0001	< 0.0001	1.47	3.80	2.58
IL-1b	pg/ml	344	262	354	529	432	357	1147	1112	634	0.0036	< 0.0001	< 0.0001	1.54	3.34	2.17
IL-8	pg/ml	939	791	639	793	664	532	1094	939	764	0.5295	0.2259	0.0348	0.85	1.17	1.38
HGF	ng/ml	0.363	0.265	0.467	0.812	0.500	0.671	2.922	2.438	2.211	0.0004	< 0.0001	< 0.0001	2.24	8.05	3.60

^#^The “ratio” and “combination” values were calculated per sample individually. As it is a ratio, there are no relevant units.

As outlined in Table 3, in the comparisons between the three groups, all biomarkers were found to be significantly different (*p* value < 0.05), except for: (i) OPG and IL-8 between the health and gingivitis groups; (ii) IL-8 between the health and periodontitis groups; and (iii) MMP-3 between the gingivitis and periodontitis groups. For quantitative analysis, we calculated the fold-change of the measured values for each biomarker (or combinations thereof) for the three examined groups (Table 3). In all cases of single biomarkers (except for TIMP-1) the ratio of the values for periodontitis/health groups was higher than the ratio of the values for gingivitis/health. Out of 10 biomarkers, 9 were differentially expressed (*p* value < 0.05) between health and periodontitis groups (with a fold change range of 0.49 to 9.69, except IL-8, whose ratio was close to 1 (1.17)); and 8 showed significant differences between health and gingivitis (with a fold change range between 0.85 and 4.97). TIMP-1 levels were significantly reduced in the periodontitis group compared to both gingivitis (0.56-fold) and health (0.49-fold) groups, while the other 9 protein levels were up-regulated in subjects with periodontitis. When the gingivitis group was compared to the periodontitis one, except MMP-3, all 9 proteins were significantly changed. The respective fold changes and *p* values are depicted in Table 3.

The relative relations (including ratios) between some of the most promising biomarkers (MMP-8 + MMP-9, MMP-8/TIMP-1, MMP-9/TIMP-1, (MMP-8 + MMP-9)/TIMP-1) were further investigated. The rationale behind choosing the markers MMP-8, MMP-9 and TIMP-1 for examining their ratios was not defined by computer modelling but was based on literature mining and biological relevance. It is known from the literature that MMP-8 and MMP-9 have similar specificity to degrade matrix, and they have the same inhibitors (e.g. TIMP-1)^3,20–22^. When comparing the gingivitis vs health groups, the values of the above biomarker relations were of the same order as the values of the single biomarkers. However, when comparing periodontitis vs health groups, the values of the aforementioned biomarker relations were significantly higher than the single biomarkers, with a magnitude up to 33.69-fold for MMP-8/TIMP-1; 27.90-fold for MMP-9/TIMP-1; 31.39-fold for (MMP-8 + MMP-9)/TIMP-1; and 8.72-fold for MMP-8 + MMP-9 (Fig. 1, Table 3). Furthermore, these values were also higher than the single biomarkers in the periodontitis group compared to the gingivitis group (*p* < 0.0001) (Table 3).

### Receiver operating characteristic curves (ROC) and their area under the curve (AUC)

**Figure 2:**
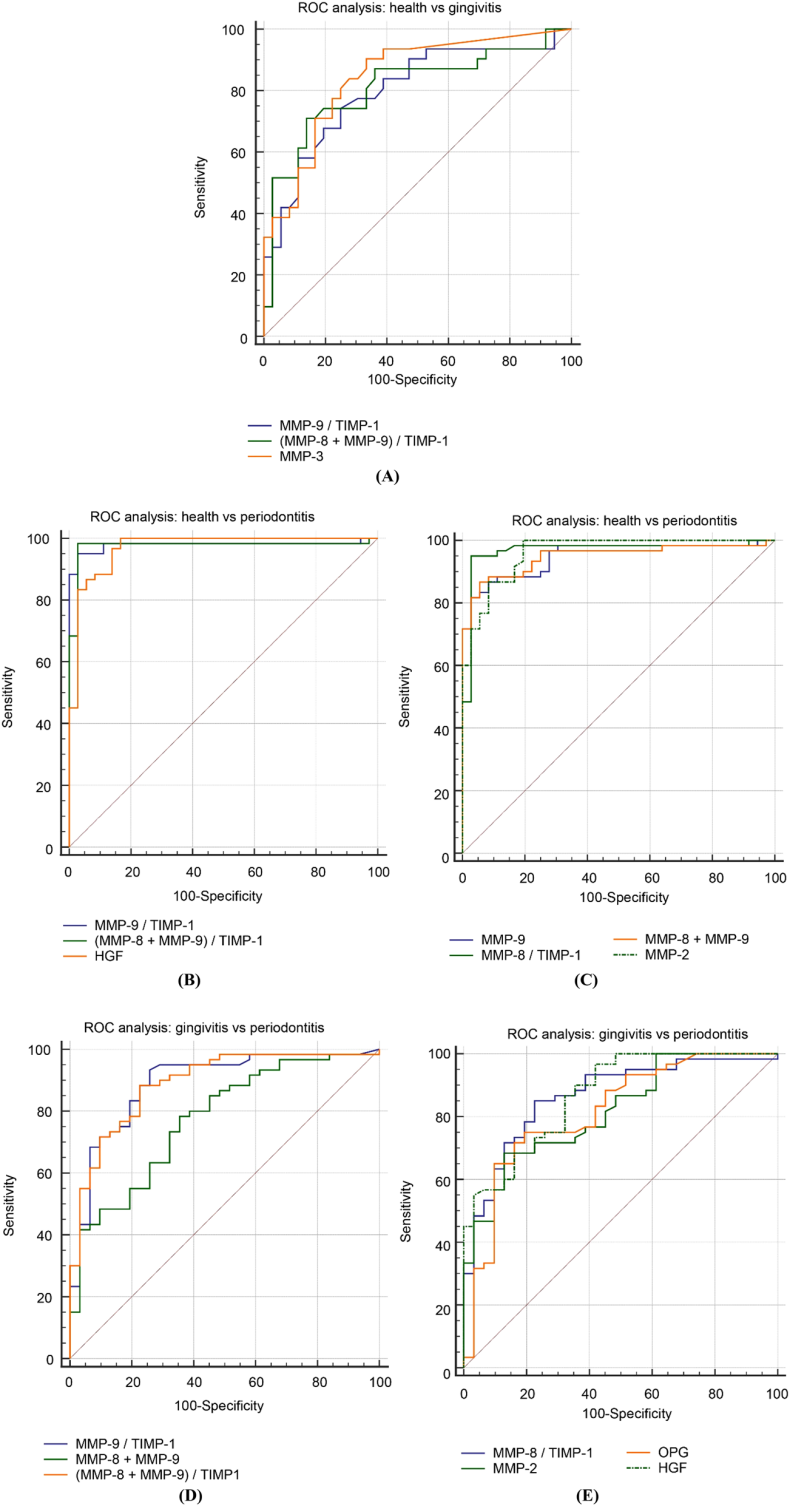
Classification and diagnostic accuracy mode for distinguishing different stages of periodontal disease. ROC (receiver-operating characteristic analysis) and AUC (the area under ROC curve) as described ealrier^3^. (**A**) health versus gingivitis (AUC ≥ 0.80); (**B**,**C**) health versus periodontitis (AUC ≥ 0.95); and (**D**,**E**) gingivitis versus periodontitis (AUC ≥ 0.85). (**B**,**C** and **D**,**E** were split in two graphs due to graphical software limitations).

We have used the ROC method and the corresponding AUC in order to assess the specificity and sensitivity to discriminate the two periodontal health states as described earlier^3^. The ROC results are given in Fig. 2. The AUC results are provided in Table 4.

**Table 4:** Area Under the Curve measurements for all biomarkers and combinations thereof.

Biomarkers or combinations thereof	Health versus gingivitis	Health versus periodontitis	Gingivitis versus periodontitis
MMP-8	0.73	0.92	0.74
MMP-9	0.78	**0.95**	0.79
TIMP-1	0.67	0.85	0.76
Ratio: MMP-8/TIMP-1	0.78	**0.97**	**0.87**
Ratio: MMP-9/TIMP-1	**0.80**	**0.98**	**0.89**
Sum: MMP-8 + MMP-9	0.76	**0.95**	**0.89**
Ratio: (MMP-8 + MMP-9)/TIMP-1	**0.81**	**0.98**	**0.89**
LBP	0.75	0.87	0.68
MMP-2	0.74	**0.96**	0.82
MMP-3	**0.84**	0.86	0.54
OPG	0.54	0.86	0.82
IL-1b	0.71	0.89	0.80
IL-8	0.55	0.57	0.64
HGF	0.75	**0.97**	**0.87**

The values above the thresholds are represented with bold numbers.

In the case of health vs gingivitis groups comparison, the ROC and corresponding AUC statistics for the selected markers and combinations thereof are presented in Fig. 2A. The plotted ROC curves showed a good predictor value of the MMP-9/TIMP-1 ratio, (MMP-8 + MMP-9)/TIMP-1 ratio, and MMP-3 for differentiation of gingivitis versus health conditions (AUC ≥ 0.80).

In the case of health vs periodontitis groups comparison, the ROC and corresponding AUC statistics for the selected markers and combinations thereof are presented in Fig. 2B,C. The plotted ROC curves showed a good predictor value of the MMP-9/TIMP-1 ratio, (MMP-8 + MMP-9)/TIMP-1 ratio, and HGF (as top three), followed by the MMP-9, MMP-2, MMP-8 + MMP-9 sum and the MMP-8/TIMP-1 ratio, for differentiation of periodontitis versus health conditions (AUC ≥ 0.95).

In the case of gingivitis vs periodontitis groups comparison, the ROC and corresponding AUC statistics for the selected markers and combinations thereof are presented in Fig. 2D,E. The plotted ROC curves showed a good predictor value for the MMP-9/TIMP-1 ratio, the (MMP-8 + MMP-9)/TIMP-1 ratio and the MMP-8 + MMP-9 sum (as top three), followed by the MMP-8/TIMP-1 ratio, HGF, MMP-2, and OPG for differentiation of periodontitis versus gingivitis conditions (AUC ≥ 0.85). It is again observed, like in the case of the unvariate statistical tests through the boxplots, that the combinations of MMP-8, MMP-9 and TIMP-1 have a significant differentiating value between the three groups.

### Classification and regression tree (CART) analysis of the selected candidates

We aimed to determine whether a statistical decision tree approach may aid in interpreting combinations of salivary markers. CART analysis was used to develop models that can classify subjects into periodontal health or disease categories. Each independent protein marker, or combinations thereof that were quantitatively measured, were inserted into the model and examined. All parameters were processed based on the internal algorithm for the derivation of the CART diagram without user influence on the selection or the order of parameters in the diagram. A split was made to maximize the sensitivity and specificity of the classification, resulting in a decision tree.

The figures resulting from the CART analysis are included in the supplementary Figure S1. The “total % of gingivitis patients” number represents the relative frequency of this parameter and is calculated by dividing the number of gingivitis patients over the total number. The same applies to the calculation of the “total % of periodontitis patients”. The resulting percentage represents the probability of gingivitis (or periodontitis) occurrence in a given group (branch in the CART diagram). The classification trees determine a set of binary if-then logical (split) conditions that permit accurate classification of the patient’s nodal status.

For discriminating health vs gingivitis groups, the predictor variables resulting from the CART analysis included MMP-9, TIMP-1 (Supplementary Figure S1(A)). When MMP-9 levels were higher than 150.3 ng/ml, no further splits were made and this biomarker, alone, can classify patients with gingivitis with an accuracy of 90.5%. When MMP-9 levels were < 150.3 ng/ml, a TIMP-1 cutoff at 124.29 ng/ml provided the next split. This split separated the healthy group with TIMP-1 concentrations > 124.29 ng/ml at an accuracy of 81.6% (31 healthy out of 38 cases that fulfilled the cutoff criteria of TIMP-1 > 124.29 ng/ml, see Supplementary S1(A)).

For discriminating health vs periodontitis groups, the predictor variables resulting from the CART analysis included HGF, MMP-9, LBP and IL-1b. Supplemenary Figure S1(B) shows the decision tree generated by the CART analysis. Taking into account the concentration levels of HGF, alone, subjects with HGF levels > 0.470 ng/ml were classified with periodontitis condition with an accuracy of 88.2%. When HGF levels were higher than 0.470 ng/ml, MMP-9 provided the most significant split at a cutoff level of 103.3 ng/ml, offering an improved discrimination accuracy of 96.4%. Additional variables, such as IL-1b and LBP increased the discriminative power of the decision tree up to 100%. The split based on IL-1b separated periodontitis patients with IL-1b levels above a cutoff level of 472 pg/ml. The split based on LBP separated periodontitis patients with LBP levels above a cutoff level of 60.0 µg/ml.

For discriminating gingivitis vs periodontitis groups, the predictor variables resulting from the CART analysis included HGF, TIMP-1, MMP-9/TIMP-1 ratio and OPG (Supplementary Figure S1(C)). At an HGF cutoff value of 0.613 pg/ml, this biomarker alone could most likely differentiate gingivitis from periodontitis at an accuracy of 79.5%. When HGF was higher than this concentration, then TIMP-1 at levels lower than 89.27 ng/ml could achieve increased identification of periodontitis cases to an accuracy of 97.2% with one false positive.

Nevertheless, even when TIMP-1 levels were > 89.27 ng/ml and HGF concentration were higher than 2.753 pg/ml, no further splits were made and the accuracy of determining periodontitis patients was as high as 94.1%. When HGF concentrations were between 0.613 and 2.753 pg/ml, OPG provided the next significant split at a cutoff level of 3.4 pmol/l. This variable at the concentrations above 3.4 pmol/l classified periodontitis group with an accuracy of 71.4%.

## Discussion

To validate a subset of existing candidate protein markers that are most relevant for periodontal disease, we performed ELISA-based analysis in a cross-sectional cohort, using saliva as a diagnostic biological fluid. During the past years, emerging protein detection technologies have revealed a large number of candidate periodontitis biomarkers^3,20^. Unfortunately, there exists a big gap between biomarker discovery and validation due to several factors^23^. Most previous studies considered a validation of a handful of proteins in saliva^9,15,24^. In the present study, we analyzed the levels of 10 candidate proteins in saliva with customized and commercially available ELISA analysis kits with either monoclonal or polyclonal antibodies. The ELISA approaches applied here were successful in reliably quantifying concentrations of the selected proteins in saliva ranging from picogram/ml to µicrogram/ml with the inter- and intra-assay CVs being < 10%, in line with earlier recommendations^25^. In order to evaluate and prioritize candidate biomarkers, we used a simple approach by combining accumulated evidence and our previously published studies rather than automated literature mining methods^26^.

Interestingly, we found that salivary levels of all candidate biomarkers with exception of IL-8 were differentially expressed (*p* < 0.05) in periodontitis cases compared to healthy individuals verifying earlier associations obtained from the literature. IL-8 is a member of the chemokine family and responsible for propagating the inflammatory response of the host during the bacterial challenge by mediating neutrophil influx into periodontal microenvironment. Although it has been shown that IL-8 has a role in periodontal disease^27^ with a clinical correlation between the severity of the disease, its level in saliva was rarely studied^28^. IL-8 in the present study did not differentiate the health versus periodontitis groups (*p* value: 0.2259) but it did differentiate the gingivitis versus periodontitis groups (*p* value: 0.0348) (Table 3). Reasons for the weak correlations could be that IL-8 is degraded by highly proteolytic bacterial enzymes in the saliva of patients with periodontitis^27,29–31^.

The least abundant protein in saliva from individuals with periodontitis was OPG. OPG levels were similar between health and gingivitis groups. OPG concentration in saliva in the periodontitis group was 3.8-fold and 2.58-fold higher compared to health and gingivitis groups, respectively, which is in line with an earlier study that detected differences in OPG in saliva from healthy and periodontitis subjects^32^. On the other hand, the demonstrated increase of OPG levels in periodontitis compared with health or gingivitis groups is in contrast with earlier studies performed in gingival crevicular fluid (GCF)^33,34^. Differences in protein levels between GCF and saliva can be expected as saliva is a cumulative pool of proteins from a different source within the oral cavity.

MMP-8 was the marker that showed the highest abundance among individuals with periodontitis. Salivary MMP-8 levels were significantly higher among patients with periodontitis compared to healthy or gingivitis cases supporting the previous studies that have demonstrated the increased collagenolytic activity in periodontal tissues which can be reflected in elevation of MMP-8 levels in oral fluids including saliva^10,35,36^. The ROC and AUC analysis for MMP-8, MMP-9 and TIMP-1 showed interesting findings: although only MMP-9, as a single biomarker, appeared able to discriminate (only) between health and periodontitis groups (Fig. 2C, Table 4), various combinations of these three biomarkers proved able to effectively discriminate: (i) between all three group comparisons, through the MMP-9/TIMP-1 and the (MMP-8 + MMP-9)/TIMP-1 ratios; and (ii) between health vs periodontitis and gingivitis vs periodontitis groups through the MMP-8/TIMP-1 ratio and the MMP-8 + MMP-9 sum. This significant discriminative power of these biomarker combinations is also evident from their absolute quantitative values in peridontitis/health and periodontitis/gingivitis measurements (Table 3). These findings agree with previous studies demonstrating that compared with healthy subjects, periodontitis or gingivitis patients exhibit higher MMP-8 in saliva and alterations in MMP-8 and TIMP-1 levels reflect changes in their relative concentration ratio^9,37,38^. An interesting finding is that the other less studied MMPs i.e. MMP-2, MMP-3 are present in saliva in concentrations by more than 100-fold lower than the MMP-8 and their levels are altered by the presence of periodontal disease^39^. This may not be surprising, since more than 25 MMPs have been identified and they do work in enzyme networks^40^. Still, our study showed that they do have an important role, as well, in discrimination between the health vs periodontitis and gingivitis vs periodontitis groups (for MMP-2) and between the health vs gingivitis groups (for MMP-3) according to the ROC analysis. These findings are in line with earlier observations that MMP-2 was higher in patients with periodontitis and treatment reduced its levels^41^. Interestingly, for the health vs gingivitis groups, MMP-3 is the only single-marker differentiator (while, interestingly, MMP-3 has a very poor discriminative role between gingivitis vs periodontitis groups, with AUC at 0.54)^42^.

CART analysis is a powerful statistical technique with which several “predictor” variables are tested to determine how they impact the “outcome” variable. It has many advantages over more traditional methods, such as multivariate regression, as it is inherently non-parametric, can handle highly skewed data and can improve the misclassification rate^43^. The CART analysis determined a panel of biomarkers including HGF, MMP-9, LBP, TIMP-1 and IL-1b that most efficiently predicted the patients with periodontitis or gingivitis with an accuracy of > 90%, even reaching 100% in some cases^44^. Numerous studies have reported the diagnostic potential of IL-1b levels in saliva, which has pro-inflammatory and bone resorption properties^36,39,45,46^. In our study, IL-1b could differentiate gingivitis and periodontitis from the health group, but also between gingivitis and periodontitis groups themselves^36,47,48^. For HGF and LBP, on the other hand, the available literature is limited for saliva. LBP is a lipopolysaccharide-binding protein, which promotes the release of cytokines in response to microbial challenge^49,50^. HGF is an antimicrobial and proinflammatory protein with a role in mucosa maintenance, and reportedly suppresses the growth of a range of bacteria^51^. More recent studies showed that higher concentrations of HGF are associated with the severe form of periodontitis^52^. In our study, LBP and HGF exhibited more than ~ 5 × higher concentration levels in periodontitis than in health groups, in line with the literature. Thus, it was shown that the results deriving from multiple biomarkers were better predictors than a single biomarker^3,11^.

The implementation and combination of results from three, rather than only one, statistical methods offers high added value to our analysis because it is known in the community that each statistical method, alone, has its strengths and weaknesses and there is no general consensus which model is the best (probably the context of use and biological knowledge drives which one to go for)^53–55^. Thus, investigators need to choose methods based on the limitations of the statistical measure, biomarker phase of development, hypothesis being tested, sample size, and clinical question. Overall, the results indicated primarily that combinations of biomarkers (among MMP-8, MMP-9, and TIMP-1) can have a significant discriminatory value and predictive power among the three groups, but also other biomarkers such as HGF, OPG, IL-1b and LBP exhibited important discriminatory value according to the CART analysis.

In conclusion, the present study has shown that the combination of high sensitivity laboratory analysis (ELISA immunoassays) with diverse powerful statistical analysis methods can reveal the ability of 10 oral biomarkers to discriminate three main groups of health, gingivitis, and periodontitis conditions, thereby providing novel insights into complex oral health conditions (gingivitis, periodontitis) that are regulated by more than one biomarkers or even combinations of them. Furthermore, the data acquisition and analysis workflow that was established in our work can be used at the next stage in order to (i) validate the examined markers in larger cohorts with diverse characteristics, where also systemic factors, such as diabetes, cardiovascular diseases will be taken into consideration^56,57^; and (ii) validate other more-recently discovered and less studied biomarkers. Results deriving from validation and verification studies like the present are important for future implementation of oral biomarkers into chair-side diagnostic systems, for the rapid and on-site characterization of the oral health condition of a patient^58,59^. Especially when these analytical techniques are combined with point-of-care diagnostic systems, as well as with digital data processing tools, the outcome can be extremely beneficial (impact-, cost-, and health-wise) for the patients, the health professionals but also the health systems.

## Methods

### Study population, clinical examination and collection of saliva samples

The conducted study was a cross-sectional study, during which 127 systemically healthy non-smoker individuals (female:male: 71:56) were recruited at the clinics of Periodontology of the faculty of dentistry, Adnan Menderes University, Aydın, Turkey. Ethical approval was given by the Ethics Committee of the School of Medicine, Ege University (ethics approval number:19–10.1 T/39), which conforms to the STROBE guidelines for observational studies^60^. All subjects participated after providing written informed consent according to the principles of the Declaration of Helsinki. Dental and medical history were compiled with dental and full-mouth periodontal examination as described earlier^61^. The full-mouth clinical periodontal examination included measurement of probing pocket depth (PPD), clinical attachment level (CAL), bleeding on probing (BOP) and plaque index (PI) at 6 sites per tooth. The periodontal diagnosis was in accordance with the criteria proposed by the 1999 classification for periodontal diseases^48,62^. The participants were categorized into four groups as follows: periodontal health (n = 36), gingivitis (n = 31), generalized aggressive periodontitis (G-AgP, n = 27) and generalized chronic periodontitis (CP, n = 33) as detailed earlier^3^. The individuals with G-AgP were systemically healthy and did not have local risk factors for periodontitis. They presented with a generalized pattern of rapid attachment and alveolar bone loss with a minimum of CAL ≥ 5 mm and PPD ≥ 6 mm on ≥ 8 teeth with radiographic bone loss ≥ 30% of root length affecting at least ≥ 3 teeth other than first molars and incisors. Individuals with generalized CP had a minimum of four non-adjacent teeth with sites with CAL ≥ 5 mm, PPD ≥ 6 mm, and ≥ 50% alveolar bone loss in at least two quadrants. The gingivitis group presented with above 50% bleeding on probing scores, but showed no CAL > 2 mm or radiographic alveolar bone loss. The healthy group had a mean BOP below 15% and had no sites with PD > 3 mm and CAL > 2 mm, and no detectable alveolar bone loss.

For further statistical analysis, CP and G-AgP subgroups have culminated into “periodontitis”. Whole unstimulated saliva samples were obtained by passive drooling into collection tubes for five min as detailed in previous work^3,48,61^. The samples were then placed on ice after collection and stored at − 80 °C until further analysis. The samples were coded and clinical information was not available to the assay performers.

### ELISA analysis

After thawing, the saliva supernatant sample from each patient was centrifuged at 4 °C for 10 min at 10,000xg and then diluted 10 times with an appropriate dilution buffer and used for the analysis of MMP-2, MMP-3, MMP-8, MMP-9, TIMP-1, OPG, LBP, IL-1b, IL-8 and HGF. Among them, the MMP-2, MMP-3, TIMP-1, OPG, LBP, IL-1b, IL-8 concentrations were determined by commercial ELISA kits (BioVendor-Laboratorní medicína a.s., Brno, Czech Republic) according to the manufacturer’s instructions^63,64^. Total MMP-8 was measured using the DuoSet ELISA Development System (Bio-Techne, Minneapolis, USA) according to manufacturer’s instructions^65^. Dedicated assays for the current study were developed for MMP-9 and HGF, and the details are given below. For the quantitative determination of MMP-9, saliva samples were diluted 1:10 with assay buffer and incubated for 1 h at room temperature in the plate with coated monoclonal anti-human MMP-9 (clone 10H8) antibody. After washing steps, specific sheep polyclonal anti-human MMP-9 HRP labelled detection antibody was added. Standards of recombinant protein MMP-9 were prepared at concentrations in the range of 0.625–50 ng/ml in assay buffer consisting of TBS-Tw-BSA-mouse IgG^64^. For the quantitative determination of HGF in human saliva, we used a highly sensitive sandwich ELISA employing mouse monoclonal anti-human HGF antibody (clone 11A9) and biotin-labelled mouse monoclonal anti-human HGF antibody (clone 9C12) provided by BioVendor—Laboratorní medicína a.s., Brno, Czech Republic. The experimental workflow followed previously-described steps by authors^44,45^. Recombinant HFG standards were produced in HEK293 cell line (BioVendor—Laboratorní medicína a.s., Brno, Czech Republic). Concentrations of 5, 2, 1, 0.5, 0.2, 0.1 and 0.05 ng/ml were prepared; 100 µl were used for the reactions. Some analytical characteristics of the HGF assay are provided: (i) Mean spiking recovery was 96%. We used two human saliva samples with baseline HGF levels of 1.4 and 2.6 ng/ml and spiked them with increasing amounts of HGF recombinant protein (+ 1, + 2 and + 5 ng/ml). (ii) Dilution linearity was 89%. We used two other human saliva samples with baseline HGF levels of 5.4 and 10.8 ng/ml for dilution linearity analysis. (iii) The LOD and inter/intra-assay CV are given in Table 2.

### Statistical analysis

Statistical power and sample size analysis was conducted on G*Power program^66^. The analysis indicated that at least 19 individuals were needed per group to have an 80% power at 0.40 f-type effect size with 0.05 Type-I error. The datasets were evaluated using three different strategies. Firstly, we used a SAS software package for statistical analysis and data visualization (SAS Institute Inc., USA). Kruskal–Wallis or Mann–Whitney tests with multiple comparisons post-hoc procedures were used to determine differences between the groups. Receiver Operating Characteristic (ROC) curves and corresponding Area Under the Curve (AUC) were derived (according to DeLong method in MedCalc (v17.11.5)) to assess the ability of single or combined proteins to discriminate between two studied health states. Moreover, an alternative statistical approach, namely Classification and Regression Tree Analysis (CART) was applied using either R statistical software or CART (Salford Systems, San Diego, USA) to determine the combination of candidate biomarkers that best predicted the selected condition^43^. Different cutoff values, based on the distributions of the markers, have been used as the potentially explanatory factors for CART analysis. The LOGISTIC procedure has been used for the CART calculation. The highest Score of Chi-square value has been used as a decision criterion (SELECTION SCORE) to determine the nodes in the CART model. Only statistically significant predictors, controlled by Chi-square test on alpha 5%, have been included into the final CART (there was no “training on the model” (use of training set) done). For the calculations of the clinical and demographic variables, Kruskal–Wallis test with Dunn´s multiple comparisions test, and chi-square test were applied (GraphPad Prism Version 8.00c for Mac OS X, GraphPad Software Inc., La Jolla California, USA). A *p* value < 0.05 was considered significant.

## Supplementary Information

Supplementary Figure S1.

## References

[1] Hajishengallis G, Lamont RJ (2012). Beyond the red complex and into more complexity: the polymicrobial synergy and dysbiosis (PSD) model of periodontal disease etiology. Mol. Oral Microbiol..

[2] Bostanci N, Bao K, Greenwood D, Silbereisen A, Belibasakis GN (2019). Periodontal disease: from the lenses of light microscopy to the specs of proteomics and next-generation sequencing. Adv. Clin. Chem..

[3] Bostanci N (2018). Targeted proteomics guided by label-free quantitative proteome analysis in saliva reveal transition signatures from health to periodontal disease. Mol. Cell Proteomics.

[4] Bostanci, N. & Belibasakis, G. N. Gingival crevicular fluid and its immune mediators in the proteomic era. *Periodontol. 2000* **76**, 68–84. 10.1111/prd.12154 (2018). 10.1111/prd.12154 29193353

[5] Giannobile, W. V. *et al.* Saliva as a diagnostic tool for periodontal disease: current state and future directions. *Periodontol. 2000* **50**, 52–64. 10.1111/j.1600-0757.2008.00288.x (2009). 10.1111/j.1600-0757.2008.00288.x PMC5695225 19388953

[6] Scannapieco FA (1994). Saliva-bacterium interactions in oral microbial ecology. Crit. Rev. Oral Biol. Med..

[7] Sexton WM (2011). Salivary biomarkers of periodontal disease in response to treatment. J. Clin. Periodontol..

[8] Grassl N (2016). Ultra-deep and quantitative saliva proteome reveals dynamics of the oral microbiome. Genome Med..

[9] Ebersole JL, Nagarajan R, Akers D, Miller CS (2015). Targeted salivary biomarkers for discrimination of periodontal health and disease(s). Front. Cell Infect. Microbiol..

[10] Silbereisen A (2020). Label-free quantitative proteomics versus antibody-based assays to measure neutrophil-derived enzymes in saliva. Proteomics Clin. Appl..

[11] Bostanci N, Bao K (2017). Contribution of proteomics to our understanding of periodontal inflammation. Proteomics.

[12] Raisanen IT (2020). A point-of-care test of active matrix metalloproteinase-8 predicts triggering receptor expressed on myeloid cells-1 (TREM-1) levels in saliva. J. Periodontol..

[13] Persson GR (1995). A multicenter clinical trial of PerioGard in distinguishing between diseased and healthy periodontal sites. (I). Study design, methodology and therapeutic outcome. J. Clin. Periodontol..

[14] Hemmings KW, Griffiths GS, Bulman JS (1997). Detection of neutral protease (Periocheck) and BANA hydrolase (Perioscan) compared with traditional clinical methods of diagnosis and monitoring of chronic inflammatory periodontal disease. J. Clin. Periodontol..

[15] Kinney JS (2011). Saliva/pathogen biomarker signatures and periodontal disease progression. J. Dent. Res..

[16] Nagarajan R, Al-Sabbagh M, Dawson D, Ebersole JL (2017). Integrated biomarker profiling of smokers with periodontitis. J. Clin. Periodontol..

[17] Liukkonen J, Gursoy UK, Pussinen PJ, Suominen AL, Kononen E (2016). Salivary concentrations of Interleukin (IL)-1beta, IL-17A, and IL-23 vary in relation to periodontal status. J. Periodontol..

[18] Paulovich AG, Whiteaker JR, Hoofnagle AN, Wang P (2008). The interface between biomarker discovery and clinical validation: The tar pit of the protein biomarker pipeline. Proteomics Clin. Appl..

[19] Rifai N, Gillette MA, Carr SA (2006). Protein biomarker discovery and validation: the long and uncertain path to clinical utility. Nat. Biotechnol..

[20] Bostanci N (2020). Salivary proteotypes of gingivitis tolerance and resilience. J. Clin. Periodontol..

[21] Akcali A (2015). Elevated matrix metalloproteinase-8 in saliva and serum in polycystic ovary syndrome and association with gingival inflammation. Innate Immun..

[22] Sorsa T (2006). Matrix metalloproteinases: contribution to pathogenesis, diagnosis and treatment of periodontal inflammation. Ann. Med..

[23] Anderson L (2014). Six decades searching for meaning in the proteome. J. Proteomics.

[24] Silbereisen A (2019). Regulation of PGLYRP1 and TREM-1 during progression and resolution of gingival inflammation. JDR Clin. Trans. Res..

[25] Jaedicke KM, Taylor JJ, Preshaw PM (2012). Validation and quality control of ELISAs for the use with human saliva samples. J. Immunol. Methods.

[26] Jordan R, Visweswaran S, Gopalakrishnan V (2014). Semi-automated literature mining to identify putative biomarkers of disease from multiple biofluids. J. Clin. Bioinforma.

[27] Belibasakis GN, Thurnheer T, Bostanci N (2013). Interleukin-8 responses of multi-layer gingival epithelia to subgingival biofilms: role of the "red complex" species. PLoS ONE.

[28] Lee A (2012). Bacterial and salivary biomarkers predict the gingival inflammatory profile. J. Periodontol..

[29] Schipper RG, Silletti E, Vingerhoeds MH (2007). Saliva as research material: biochemical, physicochemical and practical aspects. Arch. Oral Biol..

[30] Bikker FJ (2019). Salivary total protease activity based on a broad-spectrum fluorescence resonance energy transfer approach to monitor induction and resolution of gingival inflammation. Mol. Diagn. Ther..

[31] Bostanci N (2015). Secretome of gingival epithelium in response to subgingival biofilms. Mol. Oral Microbiol..

[32] Costa PP (2010). Salivary interleukin-6, matrix metalloproteinase-8, and osteoprotegerin in patients with periodontitis and diabetes. J. Periodontol..

[33] Bostanci N (2007). Differential expression of receptor activator of nuclear factor-kappaB ligand and osteoprotegerin mRNA in periodontal diseases. J. Periodontal Res..

[34] Bostanci N, Saygan B, Emingil G, Atilla G, Belibasakis GN (2011). Effect of periodontal treatment on receptor activator of NF-kappaB ligand and osteoprotegerin levels and relative ratio in gingival crevicular fluid. J. Clin. Periodontol..

[35] Akcali A (2017). Gingival inflammation and salivary or serum granulocyte-secreted enzymes in patients with polycystic ovary syndrome. J. Periodontol..

[36] Hassan MN (2018). Annexin-1 as a salivary biomarker for gingivitis during pregnancy. J. Periodontol..

[37] Sorsa, T. *et al.* Analysis of matrix metalloproteinases, especially MMP-8, in gingival creviclular fluid, mouthrinse and saliva for monitoring periodontal diseases. *Periodontol. 2000* **70**, 142–163. 10.1111/prd.12101 (2016). 10.1111/prd.12101 26662488

[38] Nascimento GG (2019). Salivary levels of MPO, MMP-8 and TIMP-1 are associated with gingival inflammation response patterns during experimental gingivitis. Cytokine.

[39] Morelli T (2014). Salivary biomarkers in a biofilm overgrowth model. J. Periodontol..

[40] Fingleton, B. Matrix metalloproteinases as regulators of inflammatory processes. *Biochim. Biophys. Acta Mol. Cell Res.* **1864** 2036–2042. 10.1016/j.bbamcr.2017.05.010 (2017). 10.1016/j.bbamcr.2017.05.010 28502592

[41] Makela M, Salo T, Uitto VJ, Larjava H (1994). Matrix metalloproteinases (MMP-2 and MMP-9) of the oral cavity: cellular origin and relationship to periodontal status. J. Dent. Res..

[42] Yilmaz M (2019). Pathogen profile and MMP-3 levels in areas with varied attachment loss in generalized aggressive and chronic periodontitis. Cent. Eur. J. Immunol..

[43] Muller R, Mockel M (2008). Logistic regression and CART in the analysis of multimarker studies. Clin. Chim. Acta.

[44] Kim YG (2016). Transcriptome sequencing of gingival biopsies from chronic periodontitis patients reveals novel gene expression and splicing patterns. Hum. Genomics.

[45] Bostanci N (2007). Interleukin-1alpha stimulation in monocytes by periodontal bacteria: antagonistic effects of Porphyromonas gingivalis. Oral Microbiol. Immunol..

[46] Ebersole JL (2013). Patterns of salivary analytes provide diagnostic capacity for distinguishing chronic adult periodontitis from health. J. Clin. Immunol..

[47] Syndergaard B (2014). Salivary biomarkers associated with gingivitis and response to therapy. J. Periodontol..

[48] Bostanci N, Ozturk VO, Emingil G, Belibasakis GN (2013). Elevated oral and systemic levels of soluble triggering receptor expressed on myeloid cells-1 (sTREM-1) in periodontitis. J. Dent. Res..

[49] Ren L, Jiang ZQ, Fu Y, Leung WK, Jin L (2009). The interplay of lipopolysaccharide-binding protein and cytokines in periodontal health and disease. J. Clin. Periodontol..

[50] Ding PH, Jin LJ (2014). The role of lipopolysaccharide-binding protein in innate immunity: a revisit and its relevance to oral/periodontal health. J. Periodontal Res..

[51] Ohshima M (2002). Hepatocyte growth factor in saliva: a possible marker for periodontal disease status. J. Oral Sci..

[52] Lonn J (2014). High concentration but low activity of hepatocyte growth factor in periodontitis. J. Periodontol..

[53] McDermott JE (2013). Challenges in biomarker discovery: combining expert insights with statistical analysis of complex omics data. Expert Opin. Med. Diagn..

[54] Hilden J (1991). The area under the ROC curve and its competitors. Med. Decis. Mak..

[55] Steyerberg EW (2012). Assessing the incremental value of diagnostic and prognostic markers: a review and illustration. Eur. J. Clin. Investig..

[56] D'Aiuto F, Orlandi M, Gunsolley JC (2013). Evidence that periodontal treatment improves biomarkers and CVD outcomes. J. Periodontol..

[57] Nylund KM (2018). Association of the salivary triggering receptor expressed on myeloid cells/its ligand peptidoglycan recognition protein 1 axis with oral inflammation in kidney disease. J. Periodontol..

[58] Mitsakakis K (2016). Chair/bedside diagnosis of oral and respiratory tract infections, and identification of antibiotic resistances for personalised monitoring and treatment. Stud. Health Technol. Inform..

[59] Taylor JJ (2019). A prototype antibody-based biosensor for measurement of salivary MMP-8 in periodontitis using surface acoustic wave technology. Sci. Rep..

[60] von Elm E (2014). The Strengthening the Reporting of Observational Studies in Epidemiology (STROBE) Statement: guidelines for reporting observational studies. Int. J. Surg..

[61] Afacan B, Ozturk VO, Emingil G, Kose T, Bostanci N (2018). Alarm anti-protease trappin-2 negatively correlates with proinflammatory cytokines in patients with periodontitis. J. Periodontol..

[62] Armitage GC (1999). Development of a classification system for periodontal diseases and conditions. Ann. Periodontol..

[63] Hassan SH, El-Refai MI, Ghallab NA, Kasem RF, Shaker OG (2015). Effect of periodontal surgery on osteoprotegerin levels in gingival crevicular fluid, saliva, and gingival tissues of chronic periodontitis patients. Dis. Markers.

[64] Costantini E (2020). Evaluation of salivary cytokines and vitamin D levels in periodontopathic patients. Int. J. Mol. Sci..

[65] Riis JL (2017). Adiponectin: Serum-saliva associations and relations with oral and systemic markers of inflammation. Peptides.

[66] Faul F, Erdfelder E, Lang AG, Buchner A (2007). G*Power 3: a flexible statistical power analysis program for the social, behavioral, and biomedical sciences. Behav. Res. Methods.

