# Supplementary material for: Validation and verification of predictive salivary biomarkers for oral health
Source: Sci Rep. 2021 Mar 19;11:6406. doi: 10.1038/s41598-021-85120-w (PMC7979790; doi:10.1038/s41598-021-85120-w)
Supplement: Supplementary file 1 — Supplementary Figure S1. [file 41598_2021_85120_MOESM1_ESM.docx]

**Validation and verification of predictive salivary biomarkers for oral health**

Nagihan Bostanci^1,*^, Konstantinos Mitsakakis^2,3^, Beral Afacan^4^, Kai Bao^1^, Benita Johannsen^2^, Desirée Baumgartner^2,3^, Lara Müller^2^, Hana Kotolová^5^, Gülnur Emingil^6^, and Michal Karpíšek ^5,7,*^

^1^Section of Periodontology and Dental Prevention, Division of Oral Diseases, Department of Dental Medicine, Karolinska Institutet, Alfred Nobels Allé 8, 14104 Huddinge, Stockholm, Sweden

^2^Hahn-Schickard, Georges-Koehler-Allee 103, 79110 Freiburg, Germany

^3^Laboratory for MEMS Applications, IMTEK – Department of Microsystems Engineering, University of Freiburg, Georges-Koehler-Allee 103, 79110 Freiburg, Germany

^4^Department of Periodontology, School of Dentistry, Aydin University, Turkey

^5^Masaryk University, Faculty of Pharmacy, Palackeho trida 1946/1, 61242 Brno, Czech Republic

^6^Department of Periodontology, School of Dentistry, Ege University, Bornova 35100, İzmir, Turkey

^7^BioVendor-Laboratorní medicína a.s., Research & Diagnostic Products Division, Karasek 1767/1, Reckovice, 62100 Brno, Czech Republic

^*^nagihan.bostanci@ki.se

^*^karpisek@biovendor.com

**Supplementary material**

|  |
| --- |
| **(A)** |
| **** |
| **(B)** |
|  |
|  |
| **(C)** |

**Figure S1.** Classification and regression tree (CART) analysis of the selected biomarkers and combinations thereof. The classification tree includes HGF (ng/ml), MMP-9/TIMP-1 ratio, TIMP-1 (ng/ml), OPG (pmol/l), MMP-9 (ng/ml), LBP (ng/ml), IL-1b (pg/ml). The root node provides the total number of samples, and the number of samples associated with health, gingivitis or periodontitis (chronic and aggressive forms combined) conditions. Each daughter node provides the respective decision rule criterion and the number of samples associated with gingivitis or periodontitis. The variable responsible for this split (together with the cut-off used) is noted below the root and the split of the overall group into two is indicated for instance by arrows. The predictor variables resulting from the CART analysis are shown for the discrimination between: (A) health vs gingivitis; (B) health vs periodontitis; and (C) gingivitis vs periodontitis groups.
